# Supplementary material for: Moiré induced organization of size-selected Pt clusters soft landed on epitaxial graphene
Source: Sci Rep. 2015 Aug 17;5:13053. doi: 10.1038/srep13053 (PMC4642513; doi:10.1038/srep13053)
Supplement: Supplementary Information [file srep13053-s1.pdf]

Moiré induced organization of size-selected Pt clusters soft landed on epitaxial graphene.

Sébastien Linas[†], Fabien Jean[‡], Tao Zhou[§], Clément Albin[†], Gilles Renaud*[§], Laurent Bardotti[†] and Florent Tournus*[†].

[†]Institut Lumière Matière (ILM), Université de Lyon, UMR5306 Université Lyon 1-CNRS, 69622 Villeurbanne, France

[‡]Institut NEEL, CNRS and Université Joseph Fourier, BP166, F-38042 Grenoble Cedex 9, France

[§]Univ. Grenoble Alpes, INAC-SP2M, F-38000 Grenoble, France
CEA, INAC-SP2M, F-38000 Grenoble, France

*Corresponding authors: Florent.tournus@univ-lyon1.fr_; gilles.renaud@cea.fr

Contents

Effect of the density of clusters. S1

Annealing from 300 to 1000 K. S3

Effect of the density of clusters.

GISAXS patterns are presented in Figure S1 for Pt clusters deposited on g/Ir(111) with medium and high density.

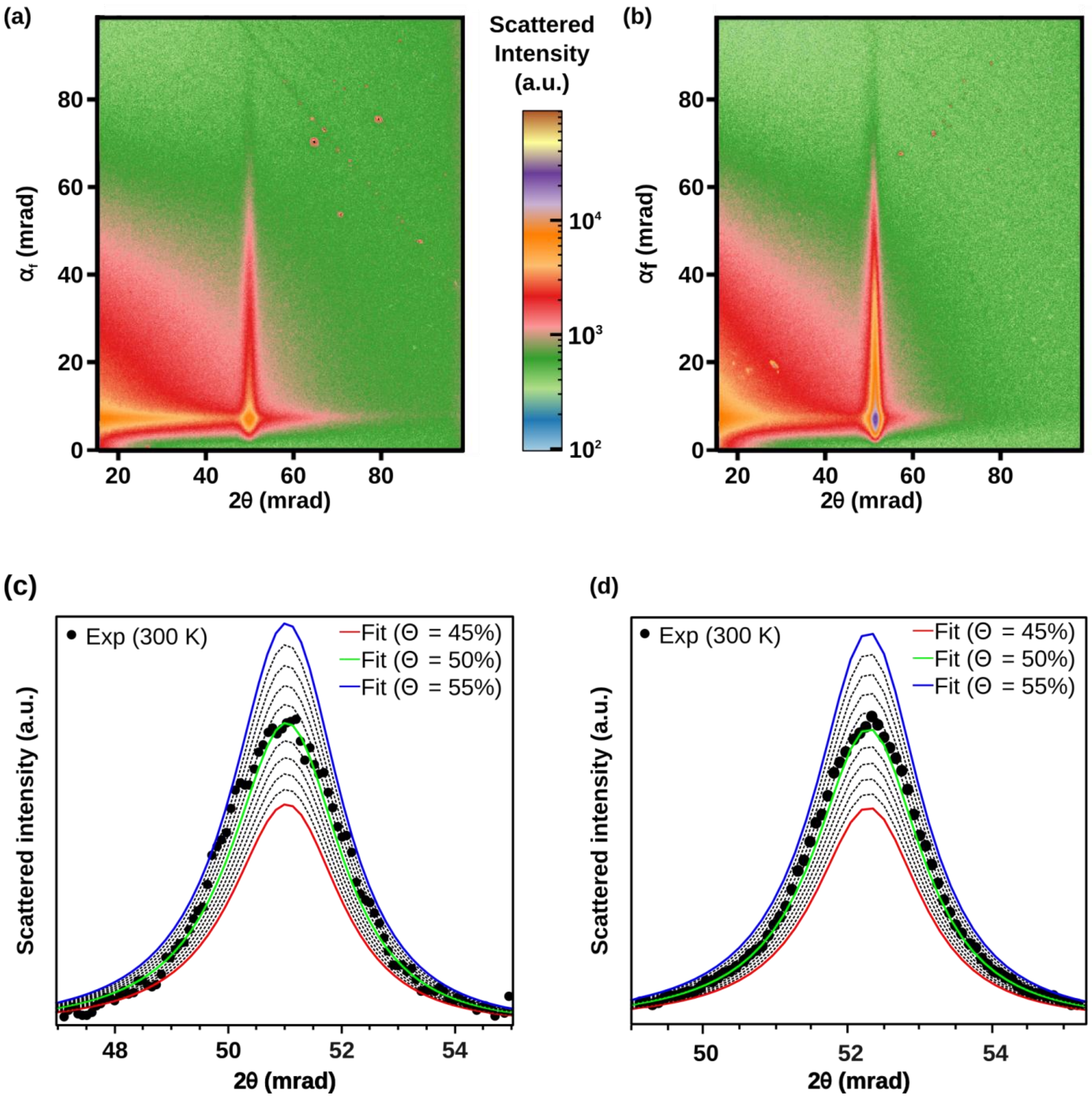

Figure S1. Pt clusters deposited on g/Ir(111) with (a,c) a medium and (b,d) a high density. (a,b) GISAXS patterns at RT with the incident beam along the Ir[100] direction. (c,d) black disks are plots of the difference between the parallel line cuts of the 2D experimental GISAXS patterns shown in (a,b) and the parallels line cuts of the corresponding GISAXS pattern with the sample azimuthally rotated by 10°. Solid lines: corresponding simulations (proportional to [$S(\boldsymbol{q})$-1]) with a proportion of NPs pinned on the moiré network (Θ) of 45% (solid red), 50% (solid green) and 55% (solid blue). The intermediates values of Θ are represented in dashed black lines, with an increment of 1%.

Annealing from 300 to 1000 K.

Parallel line cuts out of azimuth are presented in Figure S1 for various temperatures.

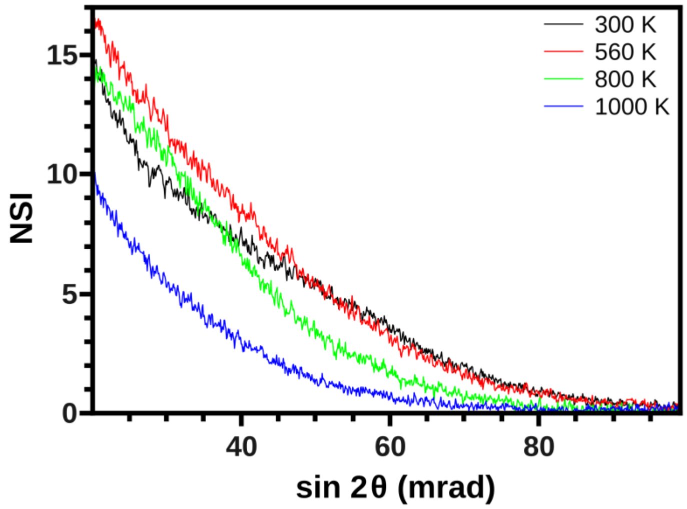

Figure S2. Parallel line cuts at RT and at $\alpha_f = \alpha_c$ = 7 mrad of the 2D GISAXS pattern with the sample azimuthally rotated by 10° for various temperatures.
